# Augmented reality and embodied learning: effects of embodiment degrees on students’ learning achievement, cognitive load, and technology acceptance

**DOI:** 10.3389/fpsyg.2025.1712261

**Published:** 2026-01-12

**Authors:** Xiaochen Suo, Baoyuan Yin, Xiaoyan Feng

**Affiliations:** 1 School of Educational Science, Harbin Normal University, Harbin, China; 2 School of Information Engineering, Henan Institute of Science and Technology, Xinxiang, China

**Keywords:** augmented reality, cognitive load, cone of experience, embodied cognition, technology acceptance

## Abstract

**Introduction:**

Embodiment in augmented reality has attracted increasing attention in educational research. This study investigated how the degree of embodied experience in augmented reality affects high school students’ learning achievement, cognitive load, and technology acceptance.

**Methods:**

Drawing on embodied cognition and the Cone of Experience, augmented reality learning tasks were designed with different degrees of embodied experience and implemented in biology instruction on cell structure. A total of 122 Chinese high school students participated and were assigned to either low- or high-embodiment augmented reality experiences. Data were analyzed using analyses of covariance, with learners’ prior knowledge scores entered as a covariate to control for pre-existing differences in knowledge level.

**Results:**

Students who engaged in high-embodiment augmented reality achieved better learning performance in both knowledge retention and transfer, and they also reported significantly lower cognitive load. In terms of technology acceptance, high-embodiment augmented reality enhanced perceived usefulness, while low-embodiment augmented reality was associated with higher perceived ease of use.

**Discussion:**

These findings demonstrate that the degree of embodiment in augmented reality experiences differentially influences learning achievement, cognitive load, and technology acceptance, offering empirical evidence and practical guidance for optimizing embodied augmented reality design in education.

## 1 Introduction

Augmented reality (AR) has emerged as a promising technology in education by extending traditional models of teaching and learning that are often constrained by time and space (Jou et al., 2025; Lu et al., 2018). By blending virtual elements with the real world and enabling mobility, AR creates new opportunities for interactive and immersive learning (Ciloglu and Ustun, 2023). In AR environments, learners can experience rich flow states, emotions, and immersive interactions (Abdul Hanid et al., 2022; Ciloglu and Ustun, 2023; Weerasinghe et al., 2022), which can motivate them to engage in active learning and knowledge construction. Beyond these general advantages, AR is particularly relevant for science subjects that involve invisible or microscopic phenomena. In high school biology, for example, students are expected to understand complex three-dimensional biology structures that are usually presented through static two-dimensional diagrams (Ciloglu and Ustun, 2023). AR can project these structures into learners’ immediate surroundings and allow them to inspect them from different perspectives, potentially making abstract content more concrete and learnable (Cai et al., 2019; O'Meara and Szita, 2021).

Embodied cognition provides a useful lens for understanding how such AR experiences may support biology learning. It posits that conceptual knowledge can be acquired and understood through bodily experiences, with cognition being grounded in sensorimotor activities (Borghi and Cimatti, 2010; Zhang et al., 2025). Embodied perspectives emphasizes bodily involvement, perceptual awareness, and the agency of the body in shaping experience (Kim and Bacos, 2020; Zahavi, 2003). High school biology teaching often deals with core ideas that are hard for students to imagine. Examples include the three-dimensional structure of cells and organelles, diffusion across membranes, and fast molecular interactions that are too small to see (Ciloglu and Ustun, 2023). Static two-dimensional diagrams alone often do not give students enough support. From an embodied cognition perspective, using hand and body movements together with external representations can help students process spatial information and rethink their ideas. It can also support the gradual construction of more connected mental models in biology learning (Castro-Alonso et al., 2024; Sullivan, 2018). In AR, learners’ actions are situated at the intersection of physical space, digital artifacts, and embodied interaction (Khazaie and Derakhshan, 2024; Mansour et al., 2025; O'Meara and Szita, 2021). For example, learners can manipulate virtual objects through gestures or full-body movements, thereby engaging in embodied participation (Bengtsson, 2013). Such participation activates neural processes linked to embodied cognition, which can enhance comprehension and knowledge internalization (Johnson-Glenberg, 2018). Recent studies in biology-related fields show that interacting with 3D or augmented molecular and cellular models helps students better understand complex structures and functions (Laubscher et al., 2024; Omurtak and Zeybek, 2022). However, AR applications do not offer a single, uniform form of embodiment. At a micro level, learners engage with gestures such as rotating, scaling, or pointing (Amara et al., 2023; Walkington et al., 2024). At a macro level, they may participate in activities involving motion control, role-playing, or tactile perception (Bos et al., 2022; Srivastava et al., 2024; Stalheim and Somby, 2024). These different forms represent varying degrees of embodiment, which can differentially influence learners’ engagement and outcomes. Although many studies acknowledge the importance of embodiment, they rarely examine how different degrees of embodiment in AR are designed, categorized, and compared. In high school biology, it therefore remains unclear how much bodily engagement, is most beneficial for learning topics such as cell structure. This study addresses this gap by focusing on degrees of embodiment in AR and asking how they should be tuned to support effective learning in secondary biology classrooms.

Learning achievement refers to the measurable outcomes of learners’ knowledge acquisition, skill development, and performance improvement (Bloom, 1956). A growing body of research indicates that AR can significantly enhance learning achievement across diverse subjects, such as science, language, and social studies, by offering interactive and multimodal learning experiences (Bursali and Yilmaz, 2019; Hu et al., 2022). The embodied nature of AR is considered a central mechanism in this improvement, as bodily interactions with virtual content can strengthen knowledge construction and retention (Johnson-Glenberg, 2018). Empirical studies have shown that embodied learning activities, such as gesture-based interactions, role-playing, and tactile manipulations, can promote deeper understanding and higher achievement scores (Pouw et al., 2014; Walkington et al., 2024). However, research also suggests that not all embodied interactions equally enhance learning achievement. In some cases, highly embodied tasks may impose excessive cognitive or physical demands, which can limit their educational effectiveness (Zou et al., 2025). This indicates that the degree of embodiment plays a critical role in shaping learning outcomes. For domains like cell biology, determining the optimal degrees of embodiment in AR experiences is therefore essential for ensuring that embodied design contributes positively to learning achievement rather than inadvertently hindering it.

Cognitive load refers to the amount of mental effort required to complete a task (Paas et al., 2003; Sweller et al., 2019). Previous studies have shown that AR can help maintain learners’ cognitive load at manageable levels (Cheng, 2016; Küçük et al., 2016; Liao et al., 2024), while others caution that the complexity of AR features may increase cognitive load (Akçayır and Akçayır, 2017). The perceptual richness afforded by embodied interactions may be a critical factor in these variations (Pouw et al., 2014). Evidence suggests that embodied agents and gestures in AR can reduce cognitive load because they externalize mental operations (Chang et al., 2022; De Melo et al., 2020). However, very highly embodied experiences, such as those in VR, can increase cognitive load (Mejia-Puig and Chandrasekera, 2023). This raises the possibility that higher degrees of embodiment in AR might similarly impose excessive demands. Current research, however, has not systematically investigated the relationship between the degree of embodiment in AR experiences and cognitive load. Understanding this relationship is essential for designing embodied AR learning that balances immersion with cognitive efficiency.

Technology acceptance models provide a robust framework for predicting users’ intentions to adopt new technologies (Davis and Davis, 1989; Yang and Yoo, 2004). Empirical research demonstrates that learners generally show positive perceptions of AR in terms of perceived usefulness (PU) and perceived ease of use (PEU) (Iqbal and Sidhu, 2022; Liu et al., 2023; Su et al., 2022). At the same time, embodied experiences themselves can shape learners’ acceptance of technology (Lin and Yu, 2024). Nevertheless, little is known about how different degrees of embodiment in AR influence learners’ willingness to adopt and engage with the technology. In school settings, where instructional time and teacher support are limited, design decisions about embodiment may affect whether students perceive AR as worth the effort required to use it. We still do not know which degrees of embodiment students are willing to use and which make AR feel too difficult or tiring. This study therefore examines which degrees of embodiment can support both positive technology acceptance and meaningful learning in high school biology.

Against this backdrop, the present study investigates how systematically varied degrees of embodiment in AR experiences affect high school students’ learning of cell structures. We designed handheld AR learning tasks with different levels of embodied experience and examined their effects on learning achievement, cognitive load, and technology acceptance. To capture a comprehensive understanding, a mixed methods approach was adopted, combining quantitative measures with qualitative interviews to both test the effects and explore learners’ subjective experiences. The findings are expected to provide empirical evidence and practical design guidelines for integrating embodiment degree into AR-based biology learning environments. Accordingly, the study addresses the following research questions:

1. What are the effects of different degrees of embodied AR experiences on learners’ learning achievement?
2. How do varying degrees of embodied AR experiences influence learners’ cognitive load?
3. In what ways do different levels of embodied AR experiences affect learners’ technology acceptance in educational contexts?

Grounded in embodied cognition and prior research on embodied and AR-supported learning, we formulated the following hypotheses to guide the quantitative analyses:

*H1a*: Learners in the high-embodiment AR experiences will demonstrate higher knowledge retention than those in the low-embodiment AR experiences.

*H1b:* Learners in the high-embodiment AR experiences will demonstrate higher transfer performance than those in the low-embodiment AR experiences.

*H2*: Learners in the high-embodiment AR experiences will report lower cognitive load than those in the low-embodiment AR experiences.

*H3a*: High-embodiment AR experiences will lead to higher PU of the lesson than low-embodiment AR experiences.

*H3b:* High-embodiment AR experiences will be associated with higher PEU than low-embodiment AR experiences.

## 2 Categorizing degrees of embodiment in AR experiences: a framework based on the cone of experience and embodied cognition

The categorization of embodiment degrees in AR experiences can be systematically grounded in two theoretical perspectives: embodied cognition and Edgar Dale’s Cone of Experience. Embodied cognition posits that perception, action, and cognition are deeply interconnected through shared neural mechanisms, with conceptual knowledge being constructed and acquired via sensorimotor experiences (Willems and Francken, 2012; Nathan, 2021). Within education, physical participation and motor activation are recognized as critical features that foster embodied cognition (Clifton et al., 2016).

The Cone of Experience, on the other hand, provides a hierarchical model of learning experiences, ranging from direct, purposeful experiences at the base to iconic and symbolic experiences at higher, more abstract levels (Dale, 1969; Subramony, 2003). When interpreted through the lens of embodied cognition, the Cone offers a framework to classify experiences according to their degree of bodily involvement. Lower-level, sensory-rich experiences correspond to a higher degree of embodiment, while upper-level, abstract symbolic experiences involve minimal bodily engagement, reflecting a lower degree of embodiment (Johnson-Glenberg et al., 2014; Nathan et al., 2014).

**Figure 1:**
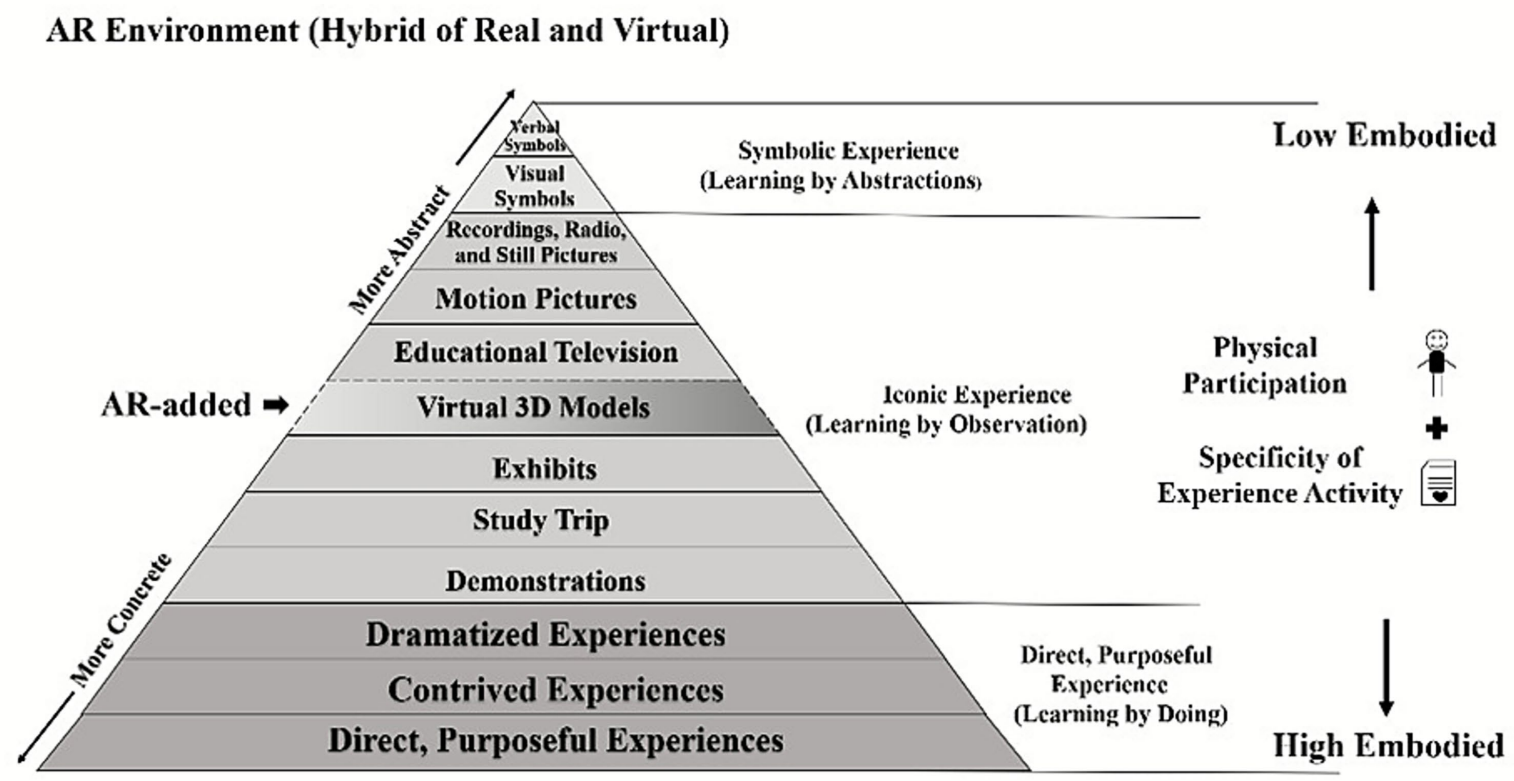
Framework of embodied experience degrees in AR based on the cone of experience and embodied cognition. Framework of embodied experience degrees in AR based on the cone of experience and embodied cognition. The diagram adapts Dale’s Cone of Experience, showing a pyramid of learning modalities from abstract to concrete. At the top are symbolic experiences (verbal, symbolic, visual symbols) representing low embodiment. Moving downward are recordings, radio, still pictures, motion pictures, educational television, and virtual 3D models—representing iconic experiences through observation. At the base are exhibits, study trips, demonstrations, dramatized experiences, contrived experiences, and direct purposeful experiences—representing high embodied, physical, and participatory learning by doing. An “AR-added” marker highlights how augmented reality integrates hybrid real and virtual environments across these layers, bridging symbolic and embodied cognition.

In AR environments, which blend real and virtual elements and provide multimodal interaction channels (Masneri et al., 2024; Chen et al., 2024), this dual-theoretical framework can be directly applied. AR affords experiences spanning all three categories of the Cone: symbolic experiences (e.g., abstract concepts represented through digital overlays or AR-based language learning, Hu et al., 2022; Bursali and Yilmaz, 2019), iconic experiences (e.g., 3D visualizations of biological structures or virtual museum explorations, Ciloglu and Ustun, 2023; Zhou et al., 2022), and direct, purposeful experiences (e.g., gesture-based anatomy practice or role-playing tasks for social learning, Karambakhsh et al., 2019; Lee, 2021). Together, these applications suggest that AR constitutes a new Cone of Experience, while still conforming to the embodiment distinctions implied in Dale’s original model, as shown in Figure 1.

Beyond categorical placement, the degree of embodiment in AR is also influenced by two key factors: (1) the level of physical participation and (2) the specificity of activity goals. Higher bodily involvement and multisensory integration increase cognitive resource allocation, strengthening embodied processing (Plass et al., 2010; Stein and Stanford, 2008). Moreover, activity specificity enables learners to form detailed neural representations of actions, facilitating proactive engagement in physical tasks (Beilock, 2015; Brucker et al., 2015).

As summarized in Figure 1, embodiment in AR is strongest at the lower levels of the Cone, weakest at the symbolic level, and moderated by the degree of physical participation and task specificity. This classification, grounded in both embodied cognition and the Cone of Experience, provides a theoretical foundation for analyzing and optimizing embodied design in AR-based learning environments. In the subsequent sections, we will build on this framework to design AR applications with varying degrees of embodiment, enabling systematic investigation of their effects on learners’ learning.

## 3 Methods

### 3.1 Research design

This study adopted a quasi-experimental research design with a between-subjects structure to examine the effects of different degrees of embodied AR experiences on high school students’ learning achievement, cognitive load, and technology acceptance. Two experimental conditions were created: a low-embodiment AR application and a high-embodiment AR application. Participants were randomly assigned to one of the two groups, with each group experiencing one type of AR application.

This study employed a quasi-experimental between-subjects design with a post-test only approach (Shadish et al., 2002). The independent variable was the degree of embodiment in AR experiences (low vs. high), and the dependent variables were learning achievement, cognitive load, and technology acceptance. Before the intervention, learners completed a prior-knowledge test on cell structures to examine baseline group differences and provide a measure for statistical control; these scores were entered as a covariate in the ANCOVA analyses to adjust for pre-existing variability (Tabachnick and Fidell, 2007). All outcome variables were assessed only after the intervention using separate instruments. In addition, semi-structured interviews were conducted with participants from both groups after the intervention to complement the quantitative results with learners’ subjective experiences of the AR activities.

### 3.2 AR applications and AR experiences

In this study, we designed two types of AR applications: low-embodiment AR application and high-embodiment AR application. Additionally, based on the analysis of the embodiment degrees of the cone of experience in AR environments, we designed the experiences in AR applications from three aspects: the levels of the Cone of Experience, physical participation, and the specificity of activity. The two AR applications were developed and implemented using Unity3D and Vuforia tools after the design was completed.

These two AR applications contain the same knowledge content, mainly targeting high school students’ learning of the abstract biological concept of “cell structure.” The content is sourced from the textbook used by first-year high school students in China (*Biology*, Compulsory Volume 1, *People’s Education Press*). To ensure the effectiveness of the AR application in actual teaching in this study, three experienced high school biology teachers participated in the design and refinement of the AR applications. Before implementation, they reviewed the materials to check the accuracy of the cell biology content, the pedagogical suitability of the learning tasks, the alignment between the embodied interactions and the learning objectives, and the overall usability of the AR interface. Based on their feedback, we revised reorganized task prompts to improve instructional coherence, and made minor interface changes to support smoother interactions. After these revisions, the experts agreed that the applications were appropriate for high school cell biology learning and consistent with curriculum requirements, which helped ensure the rigor and validity of the intervention.

The corresponding AR experiences and embodied degrees differ: the low-embodiment AR application includes rotation and zooming experiences, while the high-embodiment AR application includes haptic feedback and role-playing experiences, as shown in Table 1.

**Table 1:** Description of AR applications and AR experience embodiment degrees.

AR applications	Low-embodiment AR application		High-embodiment AR application	
Experiences	Rotation	Zooming	Haptic feedback	Role-playing
Description of the experiences	Participants tap the virtual model with one finger and slide it in a specific direction to rotate the model.	Participants use two fingers to tap the virtual model and then pinch or spread to enlarge or shrink the model.	Based on the activity instructions, when participants touched specific organelle labels on the physical AR marker, they received haptic through the device, and the corresponding virtual organelle model enlarged to provide visual feedback.	Based on the activity instructions, participants purposefully took on the role of a “cell” by matching organelle cards within the AR marker, thereby completing their own set of organelles.
Degrees of embodiment	Low	Low	High	High

When designing the low-embodiment AR application, we aimed to provide learners with experiences characterized by a lower degree of embodiment. Therefore, *iconic experience* from the lower levels of the Cone of Experience were adopted as the primary source, with no specific activities designated as experiential guidance, and learners’ physical participation was intentionally kept minimal. We incorporated the commonly observed single-finger rotation and two-finger zoom interactions in AR as the experiential components of the low-embodiment AR application in this study (Guo et al., 2022). As shown in Table 1, learners engage in these two experiences by using finger-controlled bodily actions to manipulate the virtual model’s angle and size, enabling multi-perspective observation of the cell structure.

When designing the high-embodiment AR application, we aimed to provide learners with a higher degree of embodied experience. Therefore, we adopted *direct, purposeful experiences* from the Cone of Experience as the primary source, incorporated specific activities as instructions, and ensured a higher level of physical participation. To maintain consistency in the number of experiential activities between the two AR applications, this study included haptic feedback and role-playing experiences as sources of high-embodiment experiences, as shown in Table 1.

**Figure 2:**
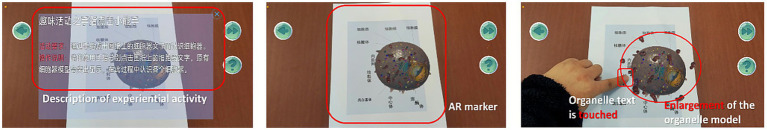
The process of haptic feedback experience. The figure illustrates three stages of interaction with an AR-enhanced learning activity. The first stage shows a description of the experiential activity displayed through augmented reality. The second stage highlights the AR marker and the associated virtual organelle model. The third stage demonstrates user interaction, where touching the organelle text triggers an enlargement of the organelle model, providing haptic feedback and deeper engagement.

Haptic feedback is an interactive experience that provides learners with a realistic sensory perception (Li et al., 2024), primarily involving vibrations or force-based feedback (Magana et al., 2019; Novak and Schwan, 2021). The haptic feedback experience in this study aims to provide learners with force-based feedback by touching specific areas of the AR marker, triggering the enlargement of corresponding virtual organelles. As shown in Figure 2, during the haptic feedback experience, learners are required to purposefully touch specific areas of the AR marker according to activity instructions, enabling them to cognitively engage with cell structures through substantial bodily movement. In this process, learners primarily gain *direct, purposeful experiences*, with clear activity objectives and a high level of physical participation. Therefore, the haptic feedback experience in this study demonstrates a high degree of embodiment.

**Figure 3:**
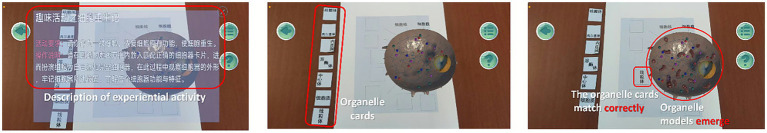
The process of role-playing experience. The figure illustrates three stages of an AR-based role-playing learning activity. The first stage shows a textual description of the experiential activity overlaid on the printed material. The second stage introduces organelle cards placed alongside the virtual organelle model. The third stage demonstrates the interactive outcome: when organelle cards are matched correctly, the corresponding organelle models emerge in augmented reality, enabling immersive role-playing and active learning.

Role-playing refers to learners achieving internal identification with the role they play through “role immersion” (Lankoski and Järvelä, 2012). It typically requires learners to “pretend” the role while thinking and acting accordingly. Embodied cognition suggests that when learners engage in role-playing, they must “simulate” the role in their minds, with this simulation based on the body’s “sensory-motor” system (Gjelsvik et al., 2018). Therefore, role-playing experience is a complex form of embodiment. As shown in Figure 3, when learners engage in role-playing, they follow specific activity instructions to purposefully play the role of a cell, adding complete organelles through direct physical activities, specifically card matching. This indicates that learners primarily gain *direct, purposeful experiences*, with clear activity objectives and high physical participation. Therefore, the embodiment degree of the role-playing experience is high.

### 3.3 Participants

To ensure the applicability of the AR-based learning content, 122 first-year (Grade 10) students from a full-time regular senior high school in China participated in the study (60 males, 62 females; M age = 15.8 years, SD = 0.6). This grade level was chosen because the official biology curriculum introduces cell structure at this stage, and students had not yet received formal, systematic instruction on this topic, although all were enrolled in the relevant biology unit. At this age, they possessed sufficient basic digital skills and, after brief training, were able to operate the AR application proficiently, making them suitable for AR-based learning of this topic. The target sample size of approximately 120 students was determined with reference to previous studies by Gu et al. (2022) and Cai et al. (2019). The study was approved by an ethics review board (Approval No. 20240906). Written consent was obtained from the school, parents, and students (participants signed individual consent forms in the presence of a research assistant to ensure understanding), and participation was voluntary. Two intact classes were then randomly selected from the Grade 10 cohort and randomly assigned to the two experimental conditions, resulting in Group 1 (*n* = 59) and Group 2 (*n* = 63), with a roughly equal gender distribution in each group. Each participant had access to a smartphone and an AR marker during the learning activities.

### 3.4 Instruments

#### 3.4.1 Demographic and prior knowledge questionnaires

In this study, we collected demographic information, including participants’ gender and age, using the questionnaire developed by Mayer and Moreno (1998). In addition, the questionnaire was used to assess participants’ familiarity with knowledge about cell. The prior knowledge questionnaire consisted of four items, each related to cell structure and animal organelles. All items were measured using a 5-point Likert scale. The total prior knowledge score was calculated by summing the scores of the four items, with each score corresponding to its respective point on the scale (e.g., 1 = 1 point, 2 = 2 points, and 5 = 5 points). The Cronbach’s alpha coefficient for the prior knowledge questionnaire was 0.711, indicating an acceptable level of reliability.

#### 3.4.2 Learning achievement questionnaires

Learning achievement was assessed with two questionnaires: a retention test and a transfer test. The retention test measured students’ ability to recall cell structure knowledge. The transfer test measured their ability to apply this knowledge in new contexts. Following prior work that used brief five-item tests in technology-enhanced learning (Li et al., 2022), both tests were designed with the guidance of three experienced high school biology teachers to ensure content validity and curricular alignment. The items were not piloted with a separate student group; instead, they were reviewed and refined together with the teachers to check clarity and suitability for Grade 10 learners. Each test contained five multiple-choice items. The KR-20 coefficients were 0.722 for the retention test and 0.796 for the transfer test, which are above the commonly accepted threshold of 0.70 for internal consistency in educational achievement measures (Tabachnick and Fidell, 2007). Scores were calculated by summing the number of correct responses on each test.

#### 3.4.3 Cognitive load scale

To assess participants’ cognitive load during AR-based learning, this study employed the cognitive load scale developed by Paas et al. (1994) to capture feedback on the perceived difficulty of using AR applications for learning. The 3 - item, 9 - point Likert - scale has a Cronbach’s alpha of 0.743, which exceeds the commonly accepted threshold of 0.70 for educational and psychological instruments (Tabachnick and Fidell, 2007). Factor analysis shows a KMO of 0.688, indicating moderate sampling adequacy according to standard guidelines (Tabachnick and Fidell, 2007). Bartlett’s Test (χ^2^ = 73.569, df = 3, *p* < 0.001), rejecting non - correlation, indicating reasonable construct validity for assessing AR - learning cognitive load. The three items of the Cognitive Load Scale are as follows:

1. How difficult do you perceive this learning task to be?
2. How much effort do you think is required to complete this learning task?
3. How challenging is it for you to understand the concepts involved in this learning task?

#### 3.4.4 Technology acceptance scale

To assess participants’ technology acceptance in AR-based learning, this study measured their acceptance from two dimensions: PU and PEU, comprising a total of 10 items. The PU measurement was adapted from Moore and Benbasat's (1991) scale on the advantages of technology use. It aimed to collect participants’ feedback on the usefulness of AR applications in learning. The scale, featuring five nine - point Likert - scale items, has a Cronbach’s alpha of 0.841 for reliability, indicating good internal consistency and exceeding the commonly recommended threshold of 0.70 for educational and psychological instruments (Tabachnick and Fidell, 2007). Factor analysis shows a KMO value of 0.786 and Bartlett’s Test results of χ^2^ = 231.129 (df = 10, p < 0.001), suggesting adequate sampling adequacy and that the correlations among the items were suitable for factor analysis. These results support the construct validity of the PU scale in this study.

The PEU measurement was adapted from Venkatesh and Davis's (2000) perceived ease of use scale. It was used to gather participants’ feedback on the ease of using AR applications for learning. The scale, featuring five nine - point Likert - scale items, has a Cronbach’s alpha of 0.745 for reliability, which also exceeds the commonly accepted threshold of 0.70 and indicates acceptable internal consistency (Tabachnick and Fidell, 2007). Factor analysis shows a KMO value of 0.766 and Bartlett’s Test results of χ^2^ = 143.51 (df = 10, *p* < 0.001) for validity, indicating that the scale demonstrates good reliability and construct validity for measuring PEU in AR - based learning. The two-dimensional scales for measuring technology acceptance demonstrated good reliability. Examples from the two scales are provided below:

1. PU: Using this learning tool improves my learning efficiency.
2. PEU: I do not expend a significant amount of mental effort when using this interaction method.

#### 3.4.5 Interview form

To complement the quantitative findings and capture learners’ subjective experiences with AR-based learning, this study employed semi-structured interviews. The interview protocol was developed based on prior literature in qualitative research design (Creswell and Poth, 2023) and was reviewed by two experts in educational technology and biology education to ensure clarity and content validity. A total of 12 participants (6 from the low-embodiment group and 6 from the high-embodiment group) were randomly selected for interviews. The sample size was considered sufficient to reach data saturation while representing both experimental conditions. The interviews were conducted 1 week after the post-test, each lasting approximately 30–40 min, and were audio-recorded with participants’ consent.

The guiding questions were designed around the three research dimensions of the study:

1. How did the AR application help you learn and understand the concept of cell structure?
2. Did you feel that using the AR application was easy or difficult, and why?
3. Would you like to use AR applications in your future learning, and what influenced your view?

### 3.5 Procedures

The experiment was conducted across 3 weeks and consisted of four main stages.

Stage 1 (Week 1): Participants first completed the demographic and prior knowledge questionnaires. Data from students with excessively high prior knowledge scores (greater than 15 points) were excluded, leaving 113 valid participants (57 in Group 1 and 56 in Group 2).

Stage 2 (Week 2): At the beginning of the experimental session, participants received a brief introduction to AR concepts and experimental guidelines. This ensured a basic understanding of AR and standardized their behavior during the subsequent activities.

Stage 3 (Week 2): After beginning, participants engaged in AR-based learning according to group assignment. Group 1 used the low-embodiment AR application (rotation and zoom), while Group 2 used the high-embodiment AR application (haptic feedback and role-playing). Each activity lasted about 15 min, followed by 15 min of integrated practice.

Stage 4 (Week 2 and Week 3): Immediately after AR learning, participants completed the learning achievement questionnaires (retention and transfer), the cognitive load scale, and the technology acceptance scale. To complement these quantitative results, semi-structured interviews were conducted with 12 randomly selected students (6 from each group) in Week 3. Each interview lasted 30–40 min and provided qualitative insights into learners’ experiences, perceptions, and acceptance of embodied AR learning.

### 3.6 Data analysis

Data were analyzed using SPSS 26.0. Before the main analyses, we examined normality, and we tested homogeneity of variances using Levene’s test; the data met these assumptions. To control for the potential influence of prior knowledge in this quasi-experimental design, we conducted ANCOVA, which are recommended for adjusting pre-existing group differences (Tabachnick and Fidell, 2007). Learners’ prior knowledge scores were included as a covariate, the intervention condition (degree of embodiment in the AR experience) was treated as a fixed factor, and learning achievement, cognitive load, and technology acceptance were entered as dependent variables.

Qualitative data were transcribed verbatim and analyzed using thematic analysis (Braun and Clarke, 2006). NVivo 12 was used to assist with data organization and coding. Two researchers independently coded the transcripts and then compared their coding, which showed substantial interrater agreement (Cohen’s *κ* = 0.82). Remaining discrepancies were resolved through discussion until consensus was reached. The final themes were used to complement and interpret the quantitative findings.

## 4 Results

Before conducting the main analyses, it was necessary to verify whether the two groups were equivalent in terms of their baseline knowledge. Therefore, we used the independent samples t-test to examine differences in prior knowledge scores between Group 1 and Group 2. The results of the t-test indicated no significant difference between the two groups (*p* = 0.700 > 0.05), suggesting that participants’ prior knowledge levels were consistent, thus meeting the condition for subsequent posttest comparisons across learning achievement, cognitive load, and technology acceptance.

### 4.1 Question 1, the results of learning achievement

ANCOVA was conducted to examine the effects of the degree of embodiment in AR experiences on students’ learning achievement, with prior knowledge entered as a covariate. The results revealed significant differences between the two groups in both retention and transfer, as shown in Table 2.

**Table 2:** ANCOVA results for learning achievement based on the degree of embodiment in AR experience.

Variables	Groups	Mean	SD	Adjusted Mean	SE	F (1,110)	*p*	*η^2^_p_*
Retention	Group1	1.96	1.500	1.967	0.214	7.051	0.009**	0.060
	Group2	2.77	1.706	2.766	0.216			
Transfer	Group1	2.11	1.633	2.108	0.229	5.546	0.020 *	0.048
	Group2	2.88	1.810	2.872	0.231			

Prior knowledge was entered as a covariate in the analysis, *p < 0.05, **p < 0.01.

For retention, Group 2 obtained a significantly higher adjusted mean score (adjusted *M* = 2.769, SE = 0.215) compared to Group 1 (adjusted *M* = 1.963, SE = 0.214), *F* (1,110) = 7.051, *p* = 0.009 < 0.01, η^2^_p_ = 0.060. For transfer, Group 2 also outperformed Group 1, with an adjusted mean of 2.874 (SE = 0.231) versus 2.106 (SE = 0.229), F (1,110) = 5.546, *p =* 0.020 < 0.05, η^2^_p_ = 0.048.

In practical terms, these results mean that students in the higher-embodiment condition answered on average about 0.8 more items correctly on both the retention and transfer measures than those in the lower-embodiment condition. For a short, single AR activity embedded in a regular high school biology lesson, this difference is educationally meaningful. As it suggests that more embodied interaction can help students both remember and apply what they have learned about cell structures. These findings are consistent with embodied cognition theory, which proposes that bodily involvement and multisensory engagement in learning tasks support deeper processing and more durable understanding. At the same time, the partial eta squared values indicate small-to-moderate effects, reminding that embodiment is one contributing factor among many in complex classroom settings and that embodied AR tasks still need to be carefully designed and integrated into broader instruction. Taken together, the results provide empirical support for Research Question 1 by showing that the degree of embodiment in AR experiences has a positive, if modest, impact on learners’ achievement in high school biology, thus supporting H1a and H1b.

### 4.2 Question 2, the results of cognitive load

As shown in Table 3, after controlling for prior knowledge, the ANCOVA results revealed a significant main effect of embodiment degree on cognitive load, *F* (1,110) = 12.264, *p* = 0.003 < 0.01, with a moderate effect size (η^2^_p_ = 0.100). Specifically, students in Group 1 (low-embodiment AR, adjusted *M* = 18.099, SE = 0.586) reported significantly higher cognitive load than those in Group 2 (high-embodiment AR, adjusted *M* = 15.185, SE = 0.591). The difference between the two groups was about 3 points on the cognitive load scale used in this study.

**Table 3:** ANCOVA results for cognitive load based on the degree of embodiment in AR experience.

Variable	Group	Mean	SD	Adjusted mean	SE	*F* (1,110)	*p*	*η^2^_p_*
Cognitive Load	Group1	18.11	4.304	18.099	0.586	12.264	0.003**	0.100
	Group2	15.18	4.501	15.185	0.591			

Prior knowledge was entered as a covariate in the analysis, *p < 0.05, **p < 0.01.

The partial eta squared value of 0.10 means that, after prior knowledge is controlled, about 10% of the variance in cognitive load can be explained by the degree of embodiment. This is a moderate effect size. In practical terms, students who used the low-embodiment AR experiences needed more mental effort to complete the biology tasks. Students in the high-embodiment condition felt that the activity was more manageable. For the AR activity embedded in a regular high school biology lesson, this reduction in perceived effort is educationally meaningful. Because it suggests that well-designed embodied interactions can free up cognitive resources for understanding complex cell structures. These findings address Research Question 2 by showing that the degree of embodiment in AR experiences has a meaningful impact on learners’ cognitive load, thus supporting H2.

### 4.3 Question 3, the results of technology acceptance

The ANCOVA results indicated a significant difference in perceived usefulness (PU) between the two groups after controlling for prior knowledge, F (1,110) = 18.973, *p* < 0.001, with a moderate effect size (η^2^_p_ = 0.147). Specifically, Group 1 (adjusted *M* = 27.530, SE = 0.958) scored significantly lower on PU than Group 2 (adjusted *M* = 33.461, SE = 0.976), as shown in Table 4. The difference between the groups was about 6 points on the PU scale, which represents a moderate effect. In practical terms, students who used the high-embodiment AR experiences were more likely to feel that the activity helped them understand the biology content and was worth the effort and class time.

**Table 4:** ANCOVA results for technology acceptance based on the degree of embodiment in AR experience.

Variable	Group	Mean	SD	Adjusted mean	SE	*F* (1,110)	*p*	*η^2^_p_*
PU	Group1	27.53	7.721	27.530	0.958	18.973	<0.001	0.147
	Group2	33.46	6.628	33.461	0.967			
PEU	Group1	32.54	6.194	32.546	0.887	12.264	<0.001	0.113
	Group2	27.82	7.11	27.819	0.895			

Prior knowledge was entered as a covariate in the analysis.

The ANCOVA results for perceived ease of use (PEU) showed a significant difference between the two groups after controlling for prior knowledge, F (1,110) = 14.068, *p* < 0.001, with a moderate effect size (η^2^_p_ = 0.113). Specifically, Group 1 (adjusted *M* = 32.546, SE = 0.887) reported significantly higher PEU than Group 2 (adjusted *M* = 27.819, SE = 0.895), as shown in Table 4. The difference of about 5 points on the PEU scale also corresponds to a moderate effect size. In a classroom setting, this means that students found the low-embodiment AR experiences easier to operate and less effortful to use during the lesson.

Taken together, these findings address Research Question 3 and show a differentiated effect of embodiment degree on technology acceptance. High-embodiment AR experiences increase students’ perceptions that the lesson is useful for learning high school biology, whereas low-embodiment AR experiences make the system feel easier to use. For teachers, this suggests a design trade-off: more embodied AR interactions can raise perceived value, but they may also reduce ease of use and require more support or scaffolding in real classroom use, thus supporting H3a but not H3b, since ease of use was rated higher for the low-embodiment AR experiences.

### 4.4 Qualitative insights from students’ experiences

To complement the quantitative analysis, semi-structured interviews were conducted to explore learners’ subjective experiences with the AR applications. Thematic analysis identified three major themes involving learning achievement, cognitive load and technology acceptance, and these themes complement the survey and test results. To make the alignment between the qualitative themes and the quantitative outcomes more transparent, Table 5 summarizes key insights and representative quotes for each outcome variable across the two embodiment conditions.

**Table 5:** Summary of qualitative themes across outcome variables and embodiment conditions.

Themes	Low-embodiment (Group 1)	High-embodiment (Group 2)
Learning achievement	*“Rotating and zooming helped me see it from different sides, but I still had to imagine how the parts worked together.”;* *“It was useful for observation, but it felt like I was only looking at a picture, not really interacting with the cell.”*	*“When I touched the AR marker, it was like the organelle really reacted to me. I remembered it much more clearly.”;* *“Playing the role of a cell and adding organelles step by step made me understand how everything works together. It was like I became part of the cell.”*
Cognitive load	*“It was simple to zoom and rotate, but then I had to imagine what each part does, which was tiring.”;* *“I kept looking at the flat screen, and sometimes I felt my brain was working harder to build the 3D picture.”*	*“When I matched the organelle cards to the right place, I did not need to think so hard—it just made sense because I was doing it.”;* *“The instructions guided me step by step, so it felt easier, even though I was moving around more.”*
Technology acceptance	*“It was very easy to rotate and zoom, but I did not feel I learned as much—it felt more like just looking at a picture.”;* *“It’s simple, but it feels more like a tool to look at than to really learn with.”*	*“The vibration feedback was not always easy to trigger, but it really helped me focus on the organelles. I think it’s worth it even if it takes effort.”;* *“The role-playing game was a bit complicated at first, but it made the knowledge clearer, so I want to use this kind of AR again.”*

With respect to learning achievement, students in the high-embodiment group frequently emphasized that the combination of haptic feedback and role-playing activities deepened their understanding of cell structures. By contrast, students in the low-embodiment group described the experience as less immersive, although still helpful. These perspectives are consistent with the quantitative results, which showed that higher embodiment significantly improved both retention and transfer.

In terms of cognitive load, perceptions of mental effort also diverged between the groups. High-embodiment participants often reported that purposeful physical activities reduced their reliance on abstract imagination. In contrast, several low-embodiment (Group 1) participants reported greater cognitive strain. These reflections triangulate the quantitative findings, suggesting that higher embodiment reduces extraneous cognitive load, even though a few students admitted that the physical tasks, such as role-playing, occasionally distracted them.

Regarding technology acceptance, both groups expressed willingness to use AR in future learning, but their perspectives reflected the trade-off between perceived usefulness and ease of use. High-embodiment (Group 2) participants valued the educational benefits, even though they sometimes struggled with the controls. These views reflect their higher perceived usefulness but lower ease of use in the survey results. In contrast, low-embodiment (Group 1) participants described the system as easier to handle but less impactful for learning. This corresponds to their survey scores showing higher PU but lower PEU in the high-embodiment experience and the opposite pattern in the low-embodiment experience.

In sum, the qualitative findings complement the quantitative evidence. They show that high-embodiment AR experiences were perceived as more effective for learning, less mentally demanding, and more valuable for future use, even though some usability challenges were noted. Low-embodiment experiences, while easier to operate with finger gestures, were seen as limited in fostering deep understanding. Together, these insights highlight the importance of balancing embodiment levels in AR design to maximize educational benefits while maintaining usability, thereby providing richer answers to Research Questions 1, 2, and 3.

## 5 Discussion and conclusion

This study examined how the embodiment degree of AR experiences influenced students’ learning achievement, cognitive load, and technology acceptance. By comparing high- and low-embodiment AR applications that conveyed identical content but differed in the type of interaction, the findings highlight the pedagogical significance of embodiment degree as a design factor in immersive learning.

The results indicated that H1a and H1b were fully supported, with high-embodiment AR experiences significantly improving both retention and transfer compared with the low-embodiment condition, suggesting that greater bodily engagement facilitates more durable knowledge construction. The effect sizes for retention and transfer were in the small-to-moderate range, suggesting that, although not dramatic, the learning gains associated with high-embodiment AR are educationally meaningful in regular classroom settings. This supports embodied cognition theory, which emphasizes the role of sensorimotor activity in shaping conceptual understanding (Johnson-Glenberg, 2018). Students in the high-embodiment group often reported that role-playing activities made them “feel like part of the cell,” while the haptic feedback helped them “remember organelles more clearly.” These qualitative insights complement the quantitative results, suggesting that when learners actively manipulate content through purposeful actions, they move beyond passive observation and achieve deeper cognitive processing. Previous research has cautioned that over-embodiment may distract learners or increase mental effort (Mejia-Puig and Chandrasekera, 2023), possibly because intense bodily interaction was combined with limited instructional guidance. In this study, however, high-embodiment activities were clearly structured and supported with explicit cues, which may explain why they facilitated rather than hindered learning achievement. Mansour et al. (2025) also report benefits of embodied AR for science concepts, but focus on younger learners and rely on qualitative evidence, without directly measuring cognitive load or acceptance. Our quantitative findings in high school biology partly converge but also reveal trade-offs (e.g., PU–PEU), suggesting that age, subject focus, and task design may shape embodiment effects differently. Another important finding concerns cognitive load. The results indicated that H2 was supported, with learners in the high-embodiment AR experiences reporting lower cognitive load than those in the low-embodiment group and a moderate effect size, suggesting that the learning process was perceived as less demanding. This aligns with prior evidence that embodied gestures and agents can reduce extraneous cognitive load by offloading abstract reasoning into sensorimotor experience (Pouw et al., 2014; Chang et al., 2022). Students explained that physical actions such as card matching or touching specific markers “*made the task easier*” and “*reduced the need to imagine everything in the head*.” Low-embodiment participants described their experience as “mentally tiring” when they tried to reconstruct 3D structures from 2D manipulations. These findings highlight that embodiment not only enriches the learning experience but also provides a scaffold that frees cognitive resources for meaningful learning. They also suggest that the specificity of embodied activities plays a critical role. When tasks are clearly guided and goal-oriented, they enhance learners’ sense of self-efficacy (Bandura and Locke, 2003) and prevent extraneous load. This process can transform embodiment into a facilitator of cognitive efficiency. The analysis of technology acceptance revealed a more complex picture. The results indicated that H3a was supported, with high-embodiment AR experiences leading to higher perceived usefulness than low-embodiment AR experiences and showing a moderate-to-large effect. In contrast, H3b was not supported, as learners in the high-embodiment condition reported lower perceived ease of use than those in the low-embodiment condition with a moderate effect, suggesting that students viewed high-embodiment AR as more valuable for learning but less easy to use. This trade-off was evident in students’ reflections: one high-embodiment learner noted, “*It was not always easy to control, but it really helped me understand science better,*” while a low-embodiment participant remarked, “*It was simple to use, but I felt like I was just looking at pictures.*” These findings are consistent with prior studies showing that embodied interaction can enhance PU by making abstract concepts tangible (Lin and Yu, 2024), while more complex interfaces may reduce PEU (Chung et al., 2015). From a motivational perspective, the inverse relationship between PU and PEU can be understood through expectancy–value and value–cost models, which view learners’ choices as a balance between perceived value and required effort (Eccles and Wigfield, 2002; Barron and Hulleman, 2015). In our study, high-embodiment AR experience appears to raise the perceived value of the lesson while also increasing perceived cost, as indicated by lower PEU. That students in this condition still rated PU highly suggests that, in this context, perceived learning benefits can outweigh usability costs when the system is seen as genuinely supporting understanding. In addition, prior work suggests that learners’ perceived convenience of activities can influence cognitive load, with more accessible applications helping to lower it (Wang et al., 2020). Our results on technology acceptance echo recent findings by Khazaie and Derakhshan (2024), who reported that robot-based AR activities in English for Medical Purposes classes led to more positive learner perceptions of the learning environment. In our study, high-embodiment AR was also perceived as more useful (higher PU), indicating that embodied AR experiences can enhance learners’ willingness to value and adopt the technology. At the same time, the lower PEU for high-embodiment AR in our data adds nuance to their results by showing a trade-off. Increasing embodiment may strengthen perceived usefulness but can also make the system feel less easy to use, which designers need to balance in classroom practice.

The qualitative data also indicate that embodiment may support students’ metacognitive and self-regulated learning processes in AR-based biology lessons. In the high-embodiment group, students reported that enacting and manipulating the content made relations between organelles more self-evident (e.g., “*playing the role of a cell and adding organelles step by step made me understand how everything works together,*” “*when I matched the organelle cards to the right place, I did not need to think so hard—it just made sense because I was doing it*”). Illustrating metacognitive regulation through strategy adaptation. From a metacognitive perspective, the high-embodiment AR experience seems to externalize key aspects of the learning process and to provide concrete feedback cues. These features can support ongoing cycles of monitoring and regulation of one’s comprehension during learning (Azevedo and Cromley, 2004; Winne and Hadwin, 2008).

Our findings also relate to and extend existing work on embodied learning. Prior research shows that embodied interaction can deepen conceptual understanding by linking abstract content to sensorimotor experience (Johnson-Glenberg et al., 2014; Johnson-Glenberg, 2018). In our study, students in the high-embodiment experience condition achieved better retention and transfer. They also reported that acting out and physically manipulating the cell structures made the relationships between organelles clearer and more coherent. These results suggest that embodied AR can support engagement during the lesson and the construction of more integrated and durable biological concepts in classroom settings.

Our results also complement cognitive offloading accounts of embodiment proposed by Pouw et al. (2014). Learners in the low-embodiment experience condition reported that they had to work harder to imagine how the parts fit and functioned together. Learners in the high-embodiment condition reported a different experience. They described how matching, enacting and manipulating the content reduced the need for intensive mental simulation and made the material easier to understand. This pattern suggests that embodied AR can shift part of the cognitive work from internal visualization to external action. It can also support students as they monitor and regulate their understanding. In this way, our study provides empirical support for these embodied cognition accounts and extends them by showing how cognitive offloading and conceptual gains appear together in a technology-enhanced science lesson.

Taken together, the findings of this study contribute to a growing understanding of how embodiment degree shapes AR learning experiences. Beyond the theoretical implications, several design-oriented recommendations can be drawn to optimize embodied AR for educational contexts.

First, designers should include purposeful and well-structured embodied activities. Tasks such as role-playing or guided haptic feedback need clear goals and clear instructions, so that physical actions focus learners on the target concepts rather than distract them (Chang et al., 2011).

Second, AR learning environments should offer training phases and scaffolding for complex embodied interactions. Short tutorials, practice modes and step-by-step prompts can help students learn how to use markers, trigger haptic feedback and perform role-playing actions before they start the main learning task. This support allows students to spend more effort on understanding the content instead of figuring out the controls.

Third, designers need to balance embodiment with usability. High-embodiment designs improved learning performance but were sometimes harder to operate, which reflects the trade-off between usefulness and ease of use (Chung et al., 2015). Simplified gestures, fewer unnecessary interface elements and intuitive core actions can reduce control difficulty while keeping the educational benefits of embodiment.

Fourth, embodied AR should use multisensory feedback that is directly tied to learning objectives. Haptic responses and simple movements that highlight key moments in a process can strengthen understanding of abstract concepts, in line with evidence that multimodal feedback enhances perceptual accuracy and immersion (Novak and Schwan, 2021).

Finally, adaptive interfaces with flexible levels of embodiment should be considered. AR systems can provide different interaction modes so that teachers and learners can choose between lower and higher embodiment depending on prior experience and task complexity. This flexibility can keep the system accessible for beginners while still supporting richer embodied interaction for more advanced learners.

In conclusion, optimizing embodied design in AR requires a careful calibration of interaction richness, activity specificity, and usability. By aligning embodied experiences with cognitive and motivational needs, AR can serve as a powerful tool to enhance learning performance, lower cognitive load, and foster positive technology acceptance among high school students.

## 6 Limitations and recommendations

There are several limitations to this study. First, AR experiences were categorized into only two degrees of embodiment. However, AR experiences are diverse, and more nuanced forms of embodiment or disembodiment may influence learning outcomes. This simplification may have masked more fine-grained patterns and thus limits the internal validity of our conclusions about the full continuum of embodiment. Second, the findings were based on a relatively small sample of 122 high school students within a narrow age range at one school. This convenience sampling and restricted context may constrain the external validity of the results and limit their generalizability to other age groups, school types, or cultural settings. Third, this study focused exclusively on declarative knowledge of “cell structure” from high school biology textbooks. As a result, the findings may not fully transfer to procedural skills or more complex conceptual learning, which could respond differently to embodied AR designs, and future work should test these effects across additional subjects and knowledge types. Finally, we did not measure or control for other learner characteristics such as spatial ability, prior AR experience, or baseline levels of flow. These unmeasured variables may have introduced bias into the observed effects and affect the reliability of the findings. Moreover, the AR applications were delivered through handheld devices, which may reduce ecological validity compared to wearable, head-mounted AR systems that are increasingly used in classrooms. Future research should address these limitations by using larger and more diverse samples, incorporating additional learner characteristics, and comparing different AR hardware and knowledge types to better establish both the internal and external validity of embodiment effects.

## Data Availability

The raw data supporting the conclusions of this article will be made available by the authors, without undue reservation.
